# Personalized combined reconstructive surgical protocols for spastic foot and ankle deformities in adult stroke: a retrospective case series

**DOI:** 10.3389/fsurg.2026.1805482

**Published:** 2026-06-03

**Authors:** Zuobin Hao, Yunli Zhou, Xingjie Gao, Guanghui Gao, Jianye Li, Yeben Wang, Wende Wang

**Affiliations:** Orthopedic Disease Diagnosis and Treatment Center, Limb Functional Reconstruction Center, Affiliated Jinan Third People's Hospital of Jining Medical University, Jinan, Shandong, China

**Keywords:** foot and ankle deformities, functional reconstruction surgery, personalized combination, spastic hemiplegia, stroke

## Abstract

**Purpose:**

Spastic foot and ankle deformities following stroke severely impair lower limb function in adults, with conservative treatments often yielding limited and short-lived results. Drawing on established experience from pediatric cerebral palsy orthopedics, we designed personalized combined surgical protocols tailored to different deformity presentations, applied them to adult stroke, and aimed to observe the feasibility and safety of the protocol for treating such complex deformities and report preliminary functional outcomes.

**Methods:**

This single-center retrospective observational case series study included patients with post-stroke spastic hemiplegia manifesting as foot drop, varus deformity, and toe flexion spasm (claw toe deformity) between September 2023 and February 2025. The personalized functional reconstruction surgical protocol was performed based on the specific deformity, which included: (A) Achilles tendon lengthening to imporve foot drop deformity; (B) Anterior tibial tendon transfer to correct varus deformity; (C) Flexor hallucis longus(FHL) lengthening and flexor digitorum longus(FDL) tenotomy to alleviate claw toe deformity. Primary outcome measures included muscle tone assessed by the modified Ashworth scale(MAS), passive ankle range of motion(ROM), 10-meter walking time(10mWT), timed up-and-go test (TUGT), and Holden score for lower limb function.

**Results:**

The study included 24 patients (21 male, 3 female) with a mean age of 53.58 ± 10.30 years and a mean follow-up of 19.23 ± 4.10 months. At postoperative follow-up intervals of 3, 6, and 12 months, as well as at the final follow-up, muscle tone in the gastrocsoleus, FHL and FDL was significantly reduced compared to preoperative levels (*P* < 0.005). Lower limb function showed an improving trend, with increased ankle ROM and varying degrees of improvement in 10mWT, TUGT and Holden score (*P* < 0.05). Postoperative resting appearance of the foot and ankle was corrected, enhancing aesthetics. No serious complications occurred throughout the follow-up period.

**Conclusion:**

For complex spastic foot and ankle deformities in adult stroke patients, this study developed personalized combined reconstructive surgical protocols. This retrospective case series suggests that the protocol has preliminary feasibility and safety in reducing muscle tone, improving ankle mobility, and lower limb motor function.

## 1 Introduction

Post-stroke spastic hemiplegia is a complex condition with considerable heterogeneity in clinical presentation among individuals. Spasticity affecting the foot and ankle is particularly common, primarily manifesting as foot drop, varus deformity, and toe flexion spasm (claw toe deformity). Foot drop impedes toe clearance during gait, increasing the risk of falls. Varus deformity alters foot contact patterns and load distribution, which can lead to pain and joint damage. Toe flexion spasm compromises push-off force and overall balance control. Furthermore, ankle clonus can be elicited in some hemiplegic patients not only during passive manipulation but also during active movements such as walking, transferring, and rehabilitation training (1). Collectively, these impairments result in declined walking capacity, often necessitating ambulatory aids, and significantly restrict daily activities and self-care independence of patient. Conservative treatments, including botulinum toxin injections, orthoses, and physical therapy, offer certain benefits in early spasticity management. However, their efficacy is often transient and requires ongoing maintenance (2). While botulinum toxin can reduce muscle tone in the short term, it does not correct secondary tendon contractures or joint deformities caused by chronic spasticity. In contrast, functional reconstruction surgeries provide lasting biomechanical improvement through anatomical correction, yielding more durable outcomes (3).

Functional reconstruction surgeries, such as tendon lengthening, tenotomy, and tendon transfer (4–8), have demonstrated significant progress in managing foot and ankle spasticity in children with cerebral palsy. However, systematic reports on their application in adults with post-stroke spastic hemiplegia remain scarce. Drawing on the established orthopaedic experience from pediatric cerebral palsy, this study developed and applied the personalized combination of multiple reconstructive surgical protocol to treat complex foot and ankle deformities in adult stroke patients, which evolved into a comprehensive treatment srategy that has achieved preliminary clinical results. Herein, we retrospectively analyze the surgical treatment of 24 patients and report it as a case series, documenting its feasibility and preliminary functional improvement.

## 2 Materials and methods

### 2.1 General information

Clinical data from patients with post-stroke spastic hemiplegia who presented at the Limb Functional Reconstruction Center of Jinan Third People's Hospital between September 2023 and February 2025 were analyzed. The patient presented with the clinical manifestation of foot drop, varus deformity, and claw toe. The inclusion criteria as follows: disease duration > 8 months, the affected side had limited passive ankle dorsiflexion (passive dorsiflexion angle < 0°, unable to reach neutral position). All patients had received at least 6 months of conservative treatment, including conventional rehabilitation training and orthosis using, with poor response or no further improvement of the deformity, and had not received botulinum toxin injections or cryoneurolysis within the previous 6 months. Patients were excluded if they had conditions such as athetosis, extrapyramidal disorders leading to increased muscle tone, cognitive impairment, severe cardiopulmonary diseases, or uncontrolled diabetes mellitus (HbA1c > 7.5%). A total of 24 patients were included in this study, comprising 21 males and 3 females, with an age range of 34 to 72 years (mean: 53.58 ± 10.30 years). The disease duration ranged from 8 months to 11 years, with a median of 4.00(2.13, 5.25) years. All patients had a history of hypertension, and 3 were concurrently diagnosed with type II diabetes mellitus (HbA1c < 7.5%). Other clinical data are shown in Table 1.

**Table 1:** Clinical characteristics of patients (*n* = 24).

Characteristics	Value
Sex	
Male	21
Female	3
Affected side	
Right	13
Left	11
Stroke type	
Cerebral infarction	6
Cerebral hemorrhage	18
walking aids	
With cane	10
Without cane	14

### 2.2 Surgical procedures

Three types of functional reconstruction procedures were used in this study, each targeting a specific foot-ankle deformity. (A) Achilles tendon lengtheningis indicated for patients presenting with clinical foot drop caused by spasticity of the gastrocsoleus and/or contracture of the Achilles tendon. (B) Tibialis anterior tendon transfer is indicated for patients with varus deformity resulting from imbalance of muscle strength and muscle tone around the foot and ankle. (C) Flexor hallucis longus (FHL) lengthening and flexor digitorum longus (FDL) tenotomy for claw toe deformity caused by spasticity of the FHL and FDL. The combined surgical protocol was personalized based on the patient's preoperative deformity pattern: Patients with foot drop, varus deformity, and claw toe received combination A + B + C; Patients with foot drop and varus deformity received combinationA + B; Patients with foot drop and claw toe received combination A + C; Patients with varus deformity and claw toe received combination B + C. The detailed surgical procedures are described below.

**(A) Achilles Tendon Lengthening:** A 4-cm midline longitudinal incision was made over the middle third of the calf. The gastrocnemius-soleus complex (gastrocsoleus) was exposed, and the tendinous portion was transversely divided at the musculotendinous junction. An intramuscular lengthening was performed by freeing and releasing the tendon. The ankle was dorsiflexed to achieve approximately 4 cm of tendon elongation. The layers and skin were closed with interrupted sutures, and a cigarette drain was placed.
**(B) Anterior Tibial Tendon Transfer:** A 2-cm oblique incision was made over the insertion of the anterior tibial tendon on the medial aspect of the foot.The tendon insertion was fully exposed, and the medial half of the anterior tibial tendon insertion was detached. A 1-cm incision was made anterior to the ankle, superior to the tarsal tunnel, to retrieve the tendon. A 2-cm incision was then made on the dorsolateral aspect of the foot to expose the cuboid and the peroneus brevis tendon. The anterior tibial tendon was passed through a subcutaneous tunnel to the dorsolateral incision. With the ankle held in dorsiflexion and eversion, the tendon was fixed under appropriate tension to the cuboid using an bioabsorbable anchor. To reinforce the fixation, the distal end of the transferred anterior tibial tendon was woven into the peroneus brevis tendon near its insertion. The layers and skin were closed with interrupted sutures, and a cigarette drain was placed.
**(C) FHL Lengthening and FDL Tenotomy:** A 3-cm longitudinal incision was made along the medial aspect of the ankle. The FHL and FDL tendons were identified. At the musculotendinous junction, the tendinous portion of each was divided to perform an intramuscular lengthening. Passive manipulation of the hallux and toes confirmed a significant improvement in flexion spasm. The wound was closed in layers with interrupted sutures, and a cigarette drain was placed. If intraoperative inspection revealed severe degeneration and fibrosis of the FHL or FDL tendons precluding effective lengthening, a complete tenotomy was performed instead.

All surgical procedures were performed by the same team of senior orthopedic surgeons. Postoperatively, the ankle was immobilized in a U-slab plaster cast in a dorsiflexed and everted position. The cast was maintained for 1.5 months, with suture removal at 14 days. After cast removal, ankle-foot orthosis (AFO) or ankle immobilization brace was used for continued support. Subsequent rehabilitation training was carried out according to the staged protocol described in the “2.4 Postoperative Rehabilitation” section.

### 2.3 Outcome measures

#### 2.3.1 Assessment of spasticity

The muscle tone of the gastrocsoleus, FHL and FDL were measured and recorded using the modified Ashworth scale (MAS) preoperatively, and at 3 months, 6 months, 12 months postoperatively, as well as at the final follow-up.

#### 2.3.2 Assessment of lower limb function

Basic functional mobility was evaluated using timed up-and-go test (TUGT, total 6-meter round trip), the 10-meter timed walking test (10mWT), and the ability to walk as scored on Holden's graded scale (9, 10). These outcomes were primarily used to assess the final functional recovery after surgery, and therefore comparisons were made only between preoperative and final follow-up.

#### 2.3.3 Ankle ROM

As most patients lacked active ankle dorsiflexion, the maximum passive ankle range of motion (ROM) of the ankle was measured using a goniometer (11, 12). The absolute value of the maximal angle between dorsiflexion and plantarflexion was recorded. Ankle ROM was compared only between preoperative and final follow-up to reflect the final structural improvement.

#### 2.3.4 Appearance comparison

The resting appearance of the foot was documented with photographs preoperatively and at the final follow-up for comparative assessment.

#### 2.3.5 Observation of complications

Patients were monitored postoperatively for complications such as pain, surgical site infection, tendon re-rupture, joint stiffness and orthotic tolerance.

All of the above outcome measures were assessed by the operating surgeons, and each patient was evaluated by the same assessor at all time points.

### 2.4 Postoperative rehabilitation

Immediate postoperative immobilization was achieved using a U-slab plaster cast. Rehabilitation training was conducted in a staged protocol: (1) Postoperative 3 days to 2 weeks: Patients were instructed to perform stationary weight-shifting and alternate lower-limb loading exercises. The frequency was 2–4 sessions daily, each lasting 2–5 min, with gradual progression based on tolerance. (2) Postoperative 2–6 weeks (before U-slab plaster cast removal): The aforementioned weight-shifting and loading exercises were continued, increased to 4–6 sessions daily for 5–10 min each. Patients were permitted to attempt slow, short-distance ambulation (distance < 100 m). (3) Postoperative 6 weeks (after U-slab plaster cast removal): The cast was removed and replaced with an AFO or ankle immobilization brace. During daytime, AFO was worn for short-duration strength and gait training. During bed rest or at night, the AFO was replaced with an ankle immobilization brace to maintain the ankle in a neutral position. (4) Postoperative 3 months: Patients were allowed to gradually resume daily activities.

### 2.5 Statistical analysis

All statistical analyses were performed using SPSS software (version 27.0). Continuous data was first tested for normality using the Shapiro–Wilk test. Normally distributed data was presented as mean ± standard deviation (x¯ ± s), while non-normally distributed data was presented as median and interquartile range (M (Q1, Q3). Statistical comparisons of pre- and postoperative values were performed using the paired-sample t-test for ankle ROM and the Wilcoxon signed-rank test for 10mWT, TUGT, and Holden score. For the above comparisons *P* < 0.05 was considered statistically significant. For MAS scores at five time points (preoperatively, and at 3 months, 6 months, 12 months postoperatively, as well as at the final follow-up), Friedman test was first used for overall comparison. If *P* < 0.05, pairwise comparisons between each pair of time points (10 comparisons in total) were further performed using the Wilcoxon signed-rank test. The Bonferroni correction was applied to adjust the significance level, with a corrected significance level of *P* < 0.005.

## 3 Results

All 24 patients underwent surgery successfully. The postoperative follow-up period ranged from 12 to 30 months, with a mean follow-up of 19.23 ± 4.10 months. During follow-up, one patient was lost to follow-up after 6 months due to a recurrent stroke, and another was lost after 8 months due to a traumatic injury to the ipsilateral limb from an accidental fall. The remaining 22 patients completed continuous follow-up. The personalized combined surgical protocol was applied based on the different deformity presentation of foot dysfunction.The A + B + C combination was used in 14 patients with significant foot drop, varus deformity, and claw toe. The A + B combination was used in 6 patients with notable foot drop and varus deformity but without significant claw toe. The A + C combination was used in 2 patients with marked foot drop and claw toe. The B + C combination was used in 2 patients presenting with significant varus deformity and claw toe (Table 2).

**Table 2:** Deformity type and personalized combined surgical protocols.

Deformity type	Combined surgical protocol*	Number
Foot drop + Varus deformity + Claw toe	A + B + C	14
Foot drop + Varus deformity	A + B	6
Foot drop + Claw toe	A + C	2
Varus deformity + Claw toe	B + C	2
Total		24

* A. Achilles Tendon Lengthening. B. Anterior Tibial Tendon Transfer. C. FHL Lengthening and FDL Tenotomy.

All patients in this study received the aforementioned combined surgical protocols. To observe the changes in muscle tone, we assessed MAS at five time points (preoperatively, and 3 months, 6 months, 12 months postoperatively, as well as final follow-up). MAS scores in gastrocsoleus, FHL and FDL showed improvement at all postoperative time points compared to preoperative levels (all *P* < 0.005) (Table 3). At the 3-month follow-up, a marked reduction in muscle tone was already observed: the gastrocsoleus decreased from 4 (3, 4) to 1 (1, 1); FHL decreased from 3 (1, 4) to 0 (0, 0); and FDL decreased from 3 (1, 4) to 0 (0, 0). This improvement remained stable in 6-month follow-up showing no significant change from the 3-month to the 6-month evaluation (*P* > 0.005). However, a partial rebound in muscle tone was noted during long-term follow-up. At the 12-month and final follow-up, the gastrocsoleus increased from 1 (1, 1) to 1 (1, 2). For the FHL and FDL, the scores reached their nadir of 0 (0, 0) at 3 months, showing a mild increase to 0.5 (0, 1) at the 12-month and final follow-up. Although these increases were not statistically significant compared to the 6-month scores (*P* > 0.005), they may suggesting a potential trend for the recurrence of spasticity at the individual level.

**Table 3:** Evaluation of muscle tone and lower limb functional outcomes.

Measure	Preoperative	Postoperative			
		3 Months	6 Months	12 Months	Final Follow-up
MAS					
gastrocsoleus	4 (3, 4)	1 (1, 1)*	1 (1, 1)*	1 (1, 2)*	1 (1, 2)*
FHL	3 (1, 4)	0 (0, 0)*	0 (0, 0.25)*	0.5 (0, 1)*	0.5 (0, 1)*
FDL	3 (1, 4)	0 (0, 0)*	0 (0, 0.25)*	0.5 (0, 1)*	0.5 (0, 1)*
10mWT(s)	41.67 (25.71, 47.62)	/	/	/	24.94 (18.18, 31.25)^#^
TUGT(s)	28.73 ± 5.18	/	/	/	21.35 ± 5.49^#^
ROM(^o^)	21.73 ± 5.73	/	/	/	41.61 ± 5.90^#^
Holden	2 (2,3)	/	/	/	4 (4,4)^#^

“x¯ ± s” represents mean ± standard deviation, and “M (Q1, Q3)” represents median and interquartile range.

FHL represents flexor hallucis longus; FDL represents flexor digitorum longus.

* *P* < 0.005 compared to the preoperative value (Bonferroni-corrected). See text for other pairwise comparisons.

# *P* < 0.05 compared to the preoperative value.

**Figure 1:**
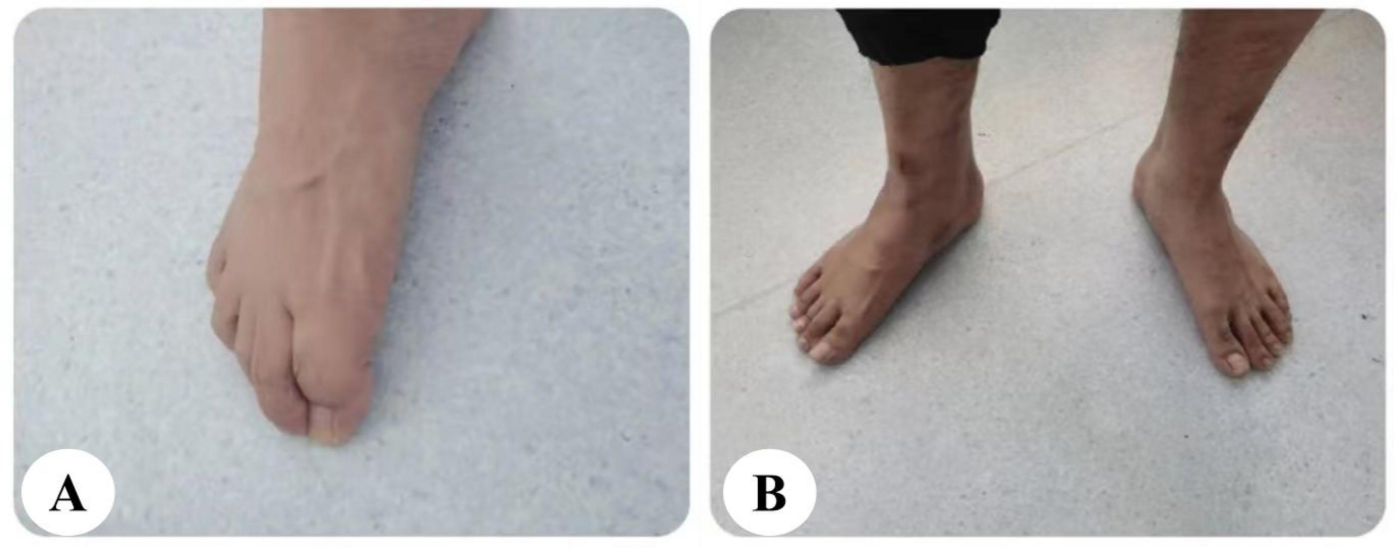
Comparative photographs show the resting appearance of the foot of a representative patient. Significant improvement is seen in the varus deformity, with the heel resting flat and resolution of the claw toe deformity. **(A)** Preoperative; **(B)** 12 months postoperative.

In addition to spasticity reduction, lower limb motor function demonstrated improvement compared to preoperative level, with all outcome measures showing statistical differences (all *P* < 0.05) (Table 3). At the final follow-up, 10mWT decreased from a preoperative 41.67 (25.71, 47.62)s to 24.94 (18.18, 31.25)s. TUGT decreased from 28.73 ± 5.18s to 21.35 ± 5.49s, indicating enhanced walking efficiency and transfer capability. Ankle ROM increased from 21.73° ± 5.73° to 41.61° ± 5.90°, reflecting improved sagittal plane mobility. Holden score also improved from 2 (2, 3) to 4 (4, 4). The patients' dependence on walking aids decreased markedly. Among the 10 patients who required a cane preoperatively, 6 were able to walk independently without aids postoperatively. The resting appearance of the foot was markedly corrected, with significant cosmetic improvement (Figure 1).

During the hospitalization period, three patients developed localized minor redness and swelling along the dorsolateral wound, which resolved completely with routine dressing changes and antisepsis. Throughout the follow-up period, no patient experienced sensory deficits, surgical site infection, tendon re-rupture, joint stiffness, or other complication. Furthermore, no overcorrection of varus deformity, deformity recurrence, revision surgery, or persistent pain was observed during follow-up. The patient could tolerate the daytime AFO (which could be fitted inside regular shoes in most cases) and the nighttime ankle immobilization brace (which maintained neutral ankle position), without significant discomfort. These observations indicate that the personalized combined surgical protocol has an overall favorable safety profile.

## 4 Disscussion

Stroke is a highly prevalent disease worldwide and ranks as the third leading cause of death. It poses a severe threat to human health and quality of life, due to its high incidence, mortality, recurrence, and disability rates, along with substantial economic burden (13). In China, approximately 3.94 million new stroke cases occur annually. While the disease burden from premature death due to stroke has declined, the burden attributable to disability continues to rise (14). Among survivors, about 70%–80% are left with varying degrees of limb dysfunction. This is a consequence of dysregulated spinal reflexes following upper motor neuron lesions.

Upper motor neurons, the primary neurons for voluntary motor control, originate from pyramidal cells in the precentral gyrus of the frontal lobe. Their axons form the corticospinal tract and other pathways, which initiate and modulate the activity of lower motor neurons and ultimately regulate somatic movement. When a stroke (including cerebral hemorrhage and infarction) damages these upper motor neurons, the regulation of spinal reflexes is disrupted. The foot, as the terminal part of the limb and influenced by abnormal neural transmission, is prone to deformities such as foot drop, varus deformity, claw toe and ankle clonus, among others (15, 16). Motor function recovery after an upper motor neuron lesion generally requires 6–9 months. Therefore, surgical options should be considered on the condition that exhaustive conservative treatment yields insufficient functional improvement (17).

In the past, predominantly exploration of functional reconstruction surgery for foot and ankle deformities had focused on children with cerebral palsy, forming various surgical techniques with mixed outcomes. For deformity caused by Achilles tendon contracture, Achilles tendon lengthening can be adopted (18, 19). It can lengthen the contracted tendon and reduce the excessive tension of the gastrocsoleus, relieves contracture-induced plantarflexion, and thereby improves foot drop deformity. This procedure carries a low risk of serious complications, and demonstrates good long-term efficacy. Varus deformity stems from an imbalance of muscular forces, where spasticity in the medial muscle groups (e.g., anterior tibial and posterior) overcomes the weakened lateral stabilizers (e.g., peroneus longus and brevis), pulling the foot inward. Anterior tibial tendon transfer is performed to correct this by relocating the tendon's insertion. This changes its mechanical action, rebalancing the inversion-eversion mechanics of the foot and correcting the deformity (5, 20). Spasticity and contracture of FHL and FDL tendons result in a flexion spasm of the toes, giving rise to pain and gait impairment. Appropriate tendon lengthening can correct this digital deformity (7, 21). Early report also existed on correcting adult claw toe through isolated or combined lengthening of the FHL and FDL (8). However, there are few reports on applying these tendon-based functional reconstruction procedures to treat complex foot and ankle deformities in adults with post-stroke spastic hemiplegia.

The key point of this study lies in drawing upon established experience from pediatric cerebral palsy orthopedics by personalizing the combination of multiple functional reconstruction surgeries for treating complex foot and ankle deformities in adults with spastic hemiplegia following stroke. Long-term spasticity following stroke often leads to the coexistence of multiple pathological changes, including tendon contractures, joint stiffness, and muscle imbalance. A single surgical procedure is insufficient to comprehensively address such complexity. Consequently, this study developed a comprehensive solution for adult complex spastic foot deformity by systematically integrating key techniques: Achilles tendon lengthening, anterior tibial tendon transfer, and flexor tendon procedures. Our results show postoperative reductions in muscle tone for the gastrocsoleus, FHL and FDL alongside increased ankle ROM and improvements in TUGT, 10mWT, and Holden score, while patient's reliance on the cane has also decreased. These results indicate that the personalized combined surgical protocol has preliminary therapeutic potential for restoring foot and ankle function in patients with post-stroke spastic hemiplegia.

The combined application of surgery in this study targets not a single deformity but restores both static and dynamic function of the foot and ankle through multidimensional adjustment of muscle balance. Achilles tendon lengthening addresses spasticity and contracture of the triceps surae muscles (medial and lateral heads of the gastrocnemius and soleus) (22). Anatomically, these muscles converge via the Achilles tendon to insert on the calcaneus, serving as the primary drivers of ankle plantar flexion. When excessively tight, they directly pull the ankle into persistent plantar flexion, resulting in foot drop. Achilles tendon lengthening can alleviate spastic tension in the triceps surae from its root cause by releasing and lengthening the contracted tendon. This reduction is directly reflected in the results, where the MAS of gastrocsoleus decreased from a preoperative 4 (3,4) to 1 (1,1). This surgical protocol releases the plantar flexion “lock” on the ankle joint, increasing passive ankle ROM from 21.73 ± 5.73° to 41.61 ± 5.90°. This change enables patient to achieve effective foot clearance during the swing phase of gait, reducing fall risk. Simultaneously, it improves tibial forward progression from mid-stance to push-off, promoting a more natural rolling gait pattern. Similarly, This also is the primary reason for the shortening of the 10mWT from 41.67(25.71, 47.62)s to 24.94(18.18, 31.25)s. However, Achilles tendon lengthening fails to correct neurogenic ankle dorsiflexor weakness or etiologically resolve true foot drop. Patients lacking active ankle dorsiflexion preoperatively may still present with foot drop postoperatively and require additional orthosis assistance and rehabilitation training. Anterior tibial tendon transfer is the cornerstone for correcting varus and restoring dynamic muscle balance. The anterior tibial tendon normally inserts on the medial cuneiform bone and base of the first metatarsal, primarily functioning for ankle dorsiflexion and foot inversion. When the peroneus longus and brevis (responsible for foot eversion) weaken, the spastic contraction of the anterior tibial loses its antagonist, leading to varus deformity. By relocating the anterior tibial to the lateral dorsum of foot (23), its insertion point is altered. This not only diminishes its inversion effect but also transforms it into an auxiliary source of dorsiflexion and eversion force, restoring normal biomechanical force lines and motion coupling in both the horizontal and coronal planes (17, 24). This study emphasized precise tensioning control to avoid over correction into valgus, embodying the principle of “balance rather than excess”. Postoperatively, all 22 patients undergoing this surgical procedure (14 A + B + C, 6 A + B, 2 B + C) demonstrated visible correction of varus deformity at rest, with the foot lying flat in a neutral position. TUGT also improved effectively, as TUGT performance highly depends on dynamic balance during turns, sit-to-stand movements. Correction of varus deformity substantially enhances stability during these activities and maintains long-term stability (25). The FHL and FDL tendons course posterior to the ankle, attaching to the distal phalanges of hallux and lesser toes, respectively. Their spasticity will cause persistent toe flexion, leading to “claw toe”. In this study, 18 patients with toe flexor spasticity, FHL lengthening and FDL tenotomy directly released the abnormal pull on the phalanges, improving forefoot contact with the ground (21). The corresponding MAS dropping from 3 (1, 4) to 0 (0, 0), demonstrating the effectiveness of this surgical procedure in resolving toe spasticity. Notably, due to the long disease duration, severe soft tissue contractures, and high muscle fibrosis in stroke patients, healing and remodeling after Achilles tendon lengthening and anterior tibial tendon transfer require strict orthotic protection to maintain biomechanical stability. However, during our long-term follow-up, we observed a partial rebound in muscle tone in some patients (Table 3). This may be related to multiple factors, including scar contracture of the reconstructed tendon in the long-term postoperative period, as well as non-compliance with strict orthotic wear and reduced adherence to rehabilitation exercises in some patients. To reduce long-term rebound, the following home-based measures are recommended: from 6 weeks postoperatively, gradual passive stretching (e.g., daily tilt-board stretching), long-term use of orthoses (daytime and nighttime), neutral ankle positioning during bed rest, family-supervised rehabilitation, and regular follow-up (online or in-person).

The personalized combination surgical protocols employed in this study may, to some extent, restore normal anatomical structure and biomechanics of the foot and ankle, and help position the limb joints in neutral or functional alignment. This creates conditions conducive to coordinated muscle group movement. In Table 3, follow-up results indicate that muscle tone significantly decreased at 3 months postoperatively (*P* < 0.005), with no significant change between 6 months and the final follow-up (*P* > 0.005). This represents a structural alteration, in stark contrast to the transient (approximately 3 months) and reversible effect produced by botulinum toxin injections (26). This stable state of low muscle tone facilitates systematic postoperative rehabilitation training. Combined with standardized rehabilitation interventions, it may contribute to improvements in limb function and Holden gait performance. Notably, we also observed that pre-existing ankle clonus in some patients decreased in frequency and intensity postoperatively but did not completely disappear. This indicates that the current surgical protocol is effective in reducing muscle tone but still has limitations in regulating neural reflex pathways, particularly the stretch reflex pathway. Our future research will further optimize surgical protocol by exploring the integration of techniques such as highly selective neurectomies (HSN) (27, 28), aiming to address ankle clonus and enhance overall therapeutic efficacy.

## 5 Conclusion

This study proposes personalized surgical combination protocols for reconstructing foot and ankle function in adult patients with post-stroke spastic hemiplegia. The protocol was performed based on the specific deformity, which included: (A) Achilles tendon lengthening to imporve foot drop deformity; (B) Anterior tibial tendon transfer to correct varus deformity; (C) FHL lengthening and FDL tenotomy to alleviate claw toe deformity. Preliminary results suggest that this protocol is feasible and safe for improving foot deformities, walking function, and quality of life in patients with spastic hemiplegia, and may provide a reference for the clinical management of such cases. However, given that this is a single-center, small-sample, uncontrolled retrospective analysis, the conclusions need to be further validated by prospective studies.

## 6 Limitations

(1) Study design limitation
  This study did not establish a control group that underwent surgery alone without any postoperative rehabilitation. Although surgery corrected the deformity and created the conditions for subsequent rehabilitation, postoperative rehabilitation training and prolonged orthotic use may have also contributed positively to functional recovery. Therefore, the relative contributions of surgery, rehabilitation, and orthotic management to the therapeutic effects cannot be determined. This is one of the major limitations of this study.
(2) Sample size and outcome measure limitations
  This is a single-center retrospective case series with a relatively small sample size, limiting the generalizability and representativeness of the results. Furthermore, the outcome measures (MAS, passive ROM, 10mWT, TUGT, Holden, photographs) have inherent limitations, for example MAS is an ordinal scale, passive ROM does not reflect active functional recovery, and photographs are descriptive. Thus, the findings should be regarded as preliminary. Future studies should expand to multi-center settings with larger sample sizes and incorporate more objective gait analysis parameters (e.g., three-dimensional gait analysis, kinetic measures).
(3) Follow-up limitation
  The follow-up period ranged from 12 to 30 months, with considerable variability across patients. Inconsistent final follow-up time points may introduce heterogeneity, affecting accurate assessment of long-term outcomes.
(4) Assessment bias
  All evaluations were performed by the operating surgeons. Despite having the same assessors for each patient, the absence of blinded outcome assessment still may introduce observer bias.

## Data Availability

The datasets generated and analyzed in this article are not publicly available due to patient privacy and confidentiality considerations but are available from the corresponding author on reasonable request and with permission from the Ethics Committee of Jinan Third People’s Hospital. Requests to access the datasets should be directed to the corresponding author.
